# Evaluation of radial distribution of cartilage degeneration and necessity of pre-contrast measurements using radial dGEMRIC in adults with acetabular dysplasia

**DOI:** 10.1186/1471-2474-13-212

**Published:** 2012-10-30

**Authors:** Li Xu, Yongbin Su, Karl-Philipp Kienle, Daichi Hayashi, Ali Guermazi, Jing Zhang, Yongming Dai, Xiaoguang Cheng

**Affiliations:** 1 Department of Radiology, 4th Medical College of Peking University (Beijing Jishuitan Hospital), 31 Xinjiekou East Street, Beijing, 100035, China; 2 Department of Radiology, Boston University School of Medicine, FGH Building 3rd Floor, 820 Harrison Avenue, Boston, MA, 02118, USA; 3 Department of Orthopedic Surgery, University of Bern, Bern, Switzerland; 4 Siemens Healthcare China, MR Collaborations NE Asia, Shanghai, 201318, China

**Keywords:** Acetabular dysplasia, Cartilage degeneration, Radial magnetic resonance imaging, dGEMRIC

## Abstract

**Background:**

The purpose of the present study was to investigate the radial distribution patterns of cartilage degeneration in dysplastic hips at different stages of secondary osteoarthritis (OA) by using radial delayed gadolinium-enhanced magnetic resonance imaging of cartilage (dGEMRIC), and to assess whether pre-contrast measurements are necessary.

**Methods:**

Thirty-five hips in 21 subjects (mean age ± SD, 27.6 ± 10.8 years) with acetabular dysplasia (lateral CE angle < 25°) were studied. Severity of OA was assessed on radiographs using Tönnis grading. Pre- (T1_pre_) and post-contrast T1 (T1_Gd_) values were measured at 7 sub-regions on radial reformatted slices acquired from a 3-dimensional (3D) T1 mapping sequence using a 1.5 T MR scanner. Values of radial T1_pre_, T1_Gd_ and ΔR1 (1/T1_Gd_ - 1/T1_pre_) of subgroups with different severity of OA were compared to those of the subgroup without OA using nonparametric tests, and bivariate linear Pearson correlations between radial T1_Gd_ and ΔR1 were analyzed for each subgroup.

**Results:**

Compared to the subgroup without OA, the subgroup with mild OA was observed with a significant decrease in T1_Gd_ in the anterosuperior to superior sub-regions (mean, 476 ~ 507 ms, *p* = 0.026 ~ 0.042) and a significant increase in ΔR1 in the anterosuperior to superoposterior and posterior sub-regions (mean, 0.93 ~ 1.37 s^-1^, *p* = 0.012 ~ 0.042). The subgroup with moderate to severe OA was observed with a significant overall decrease in T1_Gd_ (mean, 404 ~ 452 ms, *p* = 0.001 ~ 0.020) and an increase in ΔR1 (mean, 1.17 ~1.69 s^-1^, *p* = 0.001 ~ 0.020). High correlations were observed between radial T1_Gd_ and ΔR1 for all subgroups (r = −0.869 ~ −0.944, *p* < 0.001).

**Conclusions:**

Radial dGEMRIC without pre-contrast measurements is useful for evaluating different patterns of cartilage degeneration in the entire hip joint of patients with hip dysplasia, particularly for those in early stages of secondary OA.

## Background

Hip osteoarthritis (OA) is one of the major causes of musculoskeletal disability among adults [1]. One of the common anatomical predisposing factors for hip OA, acetabular dysplasia (AD), is seen not only in young patients but also in elderly patients [2]. A shallow acetabulum results in statically elevated contact pressure, reduced contact area, and joint instability, and OA tends to develop much earlier in the population with AD than in those with normal acetabular architecture [3,4]. Hip preserving procedures are desirable solutions especially for young or active patients with AD [5]. They have been widely performed and are accounted as extremely effective treatments for patients without significant secondary OA [6,7]. However, the reliability of these procedures in patients with secondary OA remains undefined [5,6,8]. The level of articular cartilage degeneration is the most important factor affecting the postoperative results [9]. Due to the unreliability of preoperative radiographic measurements, preoperative magnetic resonance imaging (MRI) is needed to assess the cartilage status in these patients [10].

Delayed gadolinium-enhanced magnetic resonance imaging of cartilage (dGEMRIC) is a minimally invasive technique to assess the biochemical properties of articular cartilage. The intravenously injected anionic contrast agent gadopentetate (Gd-DTPA^2-^) distributes in cartilage inversely to the concentration of negatively charged glycosaminoglycans (GAGs). GAGs provide cartilage with its compressive stiffness and are lost early during development of OA [11]. The dGEMRIC technique has been shown to be useful for assessing cartilage integrity in dysplastic hips by using coronal T1 mapping sequences [10,12]. The radial dGEMRIC is obtained by radial reformation from a 3D data set using dual flip angle T1 mapping sequence. Compared to conventional coronal T1 mapping, the radial dGEMRIC provides radial reformatted slices rotating from anterior to posterior perpendicular to the acetabular rim, allowing evaluation of the cartilage status in various radial regions of the entire hip joint. Distribution of the T1 dGEMRIC values measured using radial dGEMRIC have been found to be unique in different sub-groups of femoroacetabular impingement (FAI) [13]. These patterns of cartilage degeneration, reflected by the radial dGEMRIC index, have not yet been fully investigated on dysplastic hips at different stages of secondary OA.

Currently, post-contrast T1 relaxation time (T1_Gd_) is commonly used as the dGEMRIC index to determine the relative GAG levels within articular cartilage [10,12-14]. An inherent assumption behind this is that pre-contrast T1 relaxation time (T1_pre_) does not vary significantly with the health status of cartilage. However, T1_pre_ values can vary greatly in reparative cartilage and in the native cartilage with fibrillation or edema, compared to normal hyaline cartilage [15,16]. The difference between the relaxation rates (ΔR1= 1/T1_Gd_ - 1/T1_pre_) showed a better correlation with biopsy-determined GAG content in transplanted cartilage than either T1_pre_ or T1_Gd_[16]. In native cartilage, a high correlation between T1_Gd_ and ΔR1 was observed in the weight-bearing region of hip and knee joints in asymptomatic volunteers and OA patients [17-19]. The relationship between T1_Gd_ and ΔR1 on radial T1 mapping has not been reported.

Based on the above-mentioned findings, we hypothesized that: radial dGEMRIC would depict different patterns of articular cartilage degeneration in dysplastic hips at different stages of secondary OA; and that pre-contrast imaging is unnecessary for radial dGEMRIC in hips at any stage of secondary OA. The aims of this current study were to investigate, first, the radial distribution of cartilage degeneration using radial dGEMRIC indices, and second, the correlations between radial T1_Gd_ and ΔR1 in dysplastic hips at different stages of secondary OA.

## Methods

### Subjects

Subjects who were referred to our institution for a periacetabular osteotomy because of radiographically diagnosed AD (lateral center-edge angle of Wiberg, LCE angle < 25°) were recruited for this study from March to December 2010. Subjects with other hip diseases or previous hip surgery were excluded. However, subjects with closed reduction during infancy were not excluded. A total of 35 hips (16 left, 19 right) in 21 subjects (19 women, 2 men) ranging from 14 to 54 years (mean age ± SD, 27.6 ± 10.8 years) were included in this prospective study. All subjects were evaluated for clinical symptoms, and underwent both radiographic and MRI assessments. The study protocol was approved by the local ethics committee of Beijing Jishuitan Hospital and each participant signed a written informed consent before examinations.

### Clinical and radiographic assessments

Each hip was individually assessed for pain on 5 items with the Western Ontario and McMaster Universities Osteoarthritis (WOMAC) questionnaire [20], during the interval between pre- and post-contrast imaging sessions. Each item was scored on the Likert scale (0 for no pain to 4 for extreme pain). Pain score was calculated as the summed score of the 5 items for each hip.

A standing antero-posterior pelvic radiograph was performed with the beam centered on the pubic symphysis. The LCE angle, representing the severity of dysplasia, was measured as the angle formed by a vertical line through the center of the femoral head and a line connecting the center of the femoral head and the latero-superior edge of the acetabulum [21]. The minimum joint space width was measured as the minimum radial distance between the acetabulum and the femoral head in the weight-bearing zone [22]. The severity of secondary OA was determined using Tönnis grading [23]: 0, no arthritis; 1, bony sclerosis; 2, small cysts, moderate joint space narrowing; 3, large cysts, severe joint space loss, possible collapse of femoral head. Radiographic measurements were performed by an experienced musculoskeletal radiologist (YBS) using JiveX [dv] DICOM Viewer version 4.3 (VISUS Technology Transfer GmbH, Deutschland), who was blinded to the clinical information of subjects.

### MRI protocols and measurements

MRI was performed in the supine position using a 1.5 T MRI system (Espree, Siemens, Erlangen, Germany) with a body-matrix phased-array coil. For the contrast-enhanced scan, a double dose (0.4mmol/kg) of the gadolinium-based contrast agent Magnevist (Gd-DTPA^2-^, Schering, Germany) was administered intravenously. After injection all patients were asked to walk for 15 minutes and to rest for 30 minutes. The post-contrast scan was thus taken 45 minutes after injection, which is within the recommended time window for dGEMRIC [24]. The MRI protocol included: (1)axial T1-weighted (T1w) turbo spin echo (TSE) (repetition time (TR) = 491 ms, echo time (TE) = 13 ms, slice thickness/slice gap 3.0 mm/0.3 mm, field of view (FOV) 160 mm, matrix size 512 x 256, number of signal averages 1, acquisition time (TA) 4 min and 14 sec); (2) oblique coronal and sagittal proton density-weighted (PDw) TSE (TR = 3060 ms, TE = 9.1 ms, slice thickness/slice gap 2.0 mm/0.2 mm, FOV 130 mm, matrix size 256 x 205, number of signal averages 1, TA 5 min and 35 sec); (3) 3D isotropic dual-flip angle gradient echo (GRE) sequence utilizing inline T1 measurements both pre- and post-contrast (TR = 25 ms, TE = 3.6 ms, flip angles of 10° and 35°, slice thickness 0.78 mm, FOV 200 mm, matrix size 256 × 256, slab = 96, voxel size 0.78 mm^3^, TA 8 min and 46 sec). The total time for the MRI examination including patients walking and resting after contrast agent injection was between 80 and 85 min.

**Figure 1:**
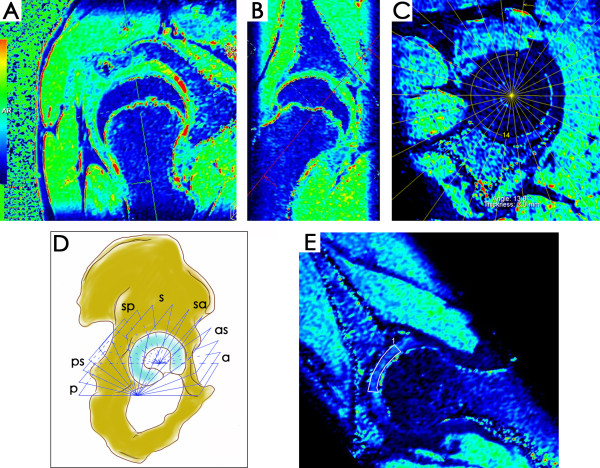
**Radial reconstruction of the T1 maps.** By alignment of the axis perpendicular to the femoral head-neck from sagittal (**A**) and coronal (**B**) views on the 3D viewer, an oblique sagittal view plane (**C**) was obtained and 13 radial slices with an interval of 13.8° were generated in the center of the femoral head. For each radial sub-regional T1_Gd_ assessment: 1, anterior; 2, anterosuperior; 3, superoanterior; 4, superior; 5, superoposterior; 6, posterosuperior and 7, posterior, 2 radial slices were included (**D**). ROI analysis was performed involving the acetabular and femoral cartilage from acetabular rim to acetabular fossa (**E**).

The 3D T1 dataset was reconstructed using a Leonardo workstation (Siemens, Erlangen, Germany). We reconstructed thirteen radial reformats rotating around the femoral head-neck axis and perpendicular to the acetabular rim, with a slice thickness of 2 mm at 13.8° intervals (Figure 1A-C). Guided by the 13 reconstructed slices, the hip joint was divided into 7 radial sub-regions: anterior, anterosuperior, superoanterior, superior, superoposterior, posterosuperior, and posterior. Each radial sub-region included 2 radial slices (Figure 1D). T1_pre_ and T1_Gd_ were measured on the 13 radial reformatted slices for each hip, with a region of interest (ROI) involving acetabular and femoral cartilage from the acetabular rim to the acetabular fossa (Figure 1E). Values of the radial T1_pre_, T1_Gd_ (mean value of the 2 slices in the same radial sub-region) and ΔR1= 1/radial T1_Gd_ - 1/radial T1_pre_ for each radial sub-region were then calculated. Global T1_pre_, T1_Gd_ and ΔR1 of each hip were calculated as the mean values of the radial indices. ROI measurements were performed by a trained radiologist (LX). To assess the intra-observer and inter-observer agreements for radial T1_pre_ and T1_Gd_ measurements, 10 hips were randomly selected one month later and radial T1_pre_ and T1_Gd_ were measured again by the same reader (LX) and another trained radiologist (YBS) on the same reformatted slices as the first measurements. Both readers were blinded to the clinical and radiographic status of the subjects.

### Statistical analyses

Statistical analyses were performed using SPSS statistical software version 11.5 (SPSS Inc., Chicago, IL, USA). The intra- and inter-observer agreements of radial T1_pre_ and T1_Gd_ measurements were evaluated on 10 random hips (7 ROIs per hip, 70 data points) using intra-class correlation (ICC) analyses.

To investigate the associations of dGEMRIC indices to the severity of lateral AD, the value of global T1_Gd_, global ΔR1, radial T1_Gd_ and radial ΔR1 was individually calculated using the Pearson correlation coefficient with LCE angle. According to the severity of secondary OA as assessed by the Tönnis grading, all hips were divided into 3 subgroups: no OA = Tönnis grade 0; mild OA = Tönnis grade 1; moderate to severe OA = Tönnis grade 2 ~ 3. Mean values and 95% confidence intervals (95% CI) of radial T1_pre_, T1_Gd_ and ΔR1 were calculated for each subgroup and 2-independent samples nonparametric tests (Mann–Whitney U test) were used to compare radial T1_pre_, T1_Gd_ and ΔR1 of subgroup 2 (Tönnis grade 1) and subgroup 3 (Tönnis grade 2 ~ 3) to those of subgroup 1 (Tönnis grade 0), respectively. To assess the necessity of pre-contrast T1 measurement of hips at different stages of secondary OA, bivariate linear Pearson correlation analysis (two-tailed) between radial T1_Gd_ and ΔR1 was individually performed for each subgroup. The significance level in this study was set at *p* < 0.05.

## Results

Results of the clinical and radiographic evaluation are summarized in Table 1.

**Table 1:** Clinical and radiographic evaluations of the study cohort

**Variables**		**Study cohort (n = 35)**
Pain Score		
(mean±SD, 4±3; range, 0~14)	0~5	28 (80%)
	6~9	5 (14%)
	≥10	2 (6%)
LCE Angle (°)		
(mean±SD, 8±12; range, -17~23)	<5	11 (31%)
	5~19	18 (51%)
	20~25	6 (17%)
Joint Space Width (mm)		
(mean±SD, 5.2±1.5; range, 2.8~10.1)	<4	6 (17%)
	4~6	21 (60%)
	>6	8 (23%)
Tönnis Grade		
	0	6 (17%)
	1	21 (60%)
	2	7 (20%)
	3	1 (3%)

### Intra- and inter-observer reliability analyses

High intra-observer correlation was detected for both radial T1_pre_ (ICC range, 0.894 ~ 0.959) and radial T1_Gd_ (ICC range, 0.813 ~ 0.928) measurements, with mean absolute differences of 52 ms (95% CI, 38 ~ 66 ms; *p* = 0.765) for the radial T1_pre_ measurement and 37 ms (95% CI, 26 ~ 47 ms; *p* = 0.319) for the radial T1_Gd_ measurement. Inter-observer correlation was also high for the radial T1_pre_ (ICC range, 0.775 ~ 0.913) and radial T1_Gd_ (ICC range, 0.787 ~ 0.918) measurements. Mean absolute difference between the 2 readers was 64 ms (95% CI, 44 ~ 83 ms; *p* = 0.358) for radial T1_pre_ measurements and 36 ms (95% CI, 27 ~ 44 ms; *p* = 0.299) for radial T1_Gd_ measurements.

### Radial distribution of dGEMRIC indices

Mean values and 95% CI of the radial and global dGEMRIC indices of the study group are shown in Table 2. Moderate correlation was detected between the LCE angle and the global T1_Gd_ (r = 0.577, *p* < 0.001) as well as radial T1_Gd_ in the weight-bearing sub-regions (superoanterior, superior, and superoposterior) (r = 0.578 ~ 0.619, *p* < 0.001) (Table 2). A similar correlation was detected between the LCE angle and the global ΔR1 (r = −0.553, *p* < 0.001) as well as radial ΔR1 in the weight-bearing sub-regions (superoanterior, superior and superoposterior) (r = −0.542 ~ −0.632, *p* < 0.001) (Table 2).

**Table 2:** Measurements of the dGEMRIC indices and the correlation coefficients between dGEMRIC indices and LCE angle

		**n = 35**							
**dGEMRIC indices**		**Radial sub-region**							**Global**
		**a**	**as**	**sa**	**s**	**sp**	**ps**	**p**	
T1_pre_	Mean (ms)	927	919	925	948	968	1021	1072	969
	95%CI (ms)	892~963	884~953	890~960	910~987	932~1004	973~1069	1007~1137	941~996
	Minimum (ms)	708	786	714	777	832	787	817	838
	Maximum (ms)	1313	1177	1179	1213	1370	1477	1783	1165
T1_Gd_	Mean (ms)	501	490	479	504	526	515	445	494
	95%CI (ms)	464~539	452~527	443~515	461~546	493~558	482~548	416~474	464~525
	Minimum (ms)	272	282	318	278	369	265	291	336
	Maximum (ms)	736	667	689	740	737	718	598	681
	Correlations^a^ with LCE, r	0.498 (*p*=0.002)	0.525 (*p*=0.001)	0.578 (*p*<0.001)	0.619 (*p*<0.001)	0.598 (*p*<0.001)	0.405 (*p*=0.016)	0.178 (*p*=0.306)	0.577 (*p*<0.001)
ΔR1	Mean (s^-1^)	1.00	1.05	1.10	1.04	0.92	1.02	1.37	1.07
	95%CI (s^-1^)	0.84~1.16	0.87~1.24	0.92~1.27	0.85~1.23	0.79~1.05	0.86~1.19	1.19~1.55	0.93~1.21
	Minimum (s^-1^)	0.33	0.40	0.21	0.10	0.31	0.29	0.55	0.45
	Maximum (s^-1^)	2.56	2.69	2.08	2.52	1.61	3.05	2.62	1.99
	Correlations^a^ with LCE, r	−0.392 (*p*=0.020)	−0.439 (*p*=0.008)	−0.542 (*p*=0.001)	−0.586 (*p*<0.001)	−0.632 (*p*<0.001)	−0.446 (*p*=0.007)	−0.219 (*p*=0.205)	−0.553 (*p*=0.001)

^*a*^Correlation was calculated as Pearson correlation coefficient.

**Figure 2:**
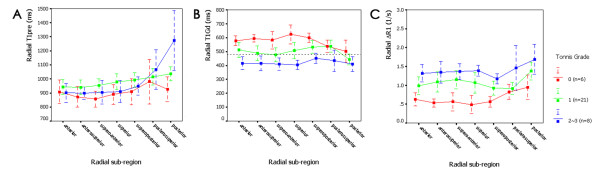
**Radial distribution of T1**_**pre**_**(A), T1**_**Gd**_**(B) and** Δ**R1 (C) according to Tönnis grading.** Compared to the Tönnis grade 0 subgroup, T1_pre_ increased significantly in the superoanterior (*p* = 0.036) and posterior (*p* = 0.022) sub-regions in the Tönnis grade 1 subgroup and in the posterior sub-region (*p* = 0.001) in the Tönnis grade 2 ~ 3 subgroup; there was a significant decrease in T1_Gd_ in the anterosuperior to superior sub-regions (*p* = 0.026 ~ 0.042) in the Tönnis grade 1 subgroup and a significant overall decrease (*p* = 0.001 ~ 0.020) in the Tönnis grade 2 ~ 3 subgroup; ΔR1 increased significantly in the anterosuperior to superoposterior (*p* = 0.012 ~ 0.042) and posterior (*p* = 0.042) sub-regions in the Tönnis grade 1 subgroup and there was a significant overall increase (*p* = 0.001 ~ 0.020) in the Tönnis grade 2 ~ 3 subgroup.

Subgroup analyses showed different patterns of dGEMRIC indices distribution in various radial sub-regions. T1_pre_ ranged from 856 to 980 ms (95% CI, 800 ~ 1140 ms) in hips without OA. Compared to that subgroup, a significant increase in T1_pre_ was detected in the superoanterior (*p* = 0.036) and posterior (*p* = 0.022) sub-regions in hips with mild OA and only in the posterior sub-region (*p* = 0.001) in hips with moderate to severe OA (Figure 2A).

Using the lower limit of the normal range of T1_Gd_ (480 ms) in articular cartilage of the hip joint as the reference standard [12], radial T1_Gd_ were found at normal levels in all sub-regions except the posterior in hips without OA, but decreased into the abnormal range in the anterior to superior sub-regions as well as in the posterior in hips with mild OA (Figure 2B). Compared to the hips without OA, a significant decrease in radial T1_Gd_ was observed in the anterosuperior (*p* = 0.042), superoanterior (*p* = 0.026) and superior (*p* = 0.031) sub-regions in hips with mild OA. In hips with moderate to severe OA, a significant overall decrease (*p* = 0.001 ~ 0.020) in radial T1_Gd_ was observed compared to the hips without OA, with an upper limit of 95% CI lower than 480 ms in most sub-regions (anterior, anterosuperior, superoanterior, superior, posterior) (Figure 2B).

The mean value of radial ΔR1 ranged from 0.49 to 0.95 s^-1^ (95% CI, 0.25 ~ 1.27 s^-1^) in hips without OA, 0.92 to 1.37 (95% CI, 0.74 ~ 1.61 s^-1^) in hips with mild OA, and 1.17 to 1.69 s^-1^ (95% CI, 0.86 ~ 2.08 s^-1^) in hips with moderate to severe OA (Figure 2C). Compared to the hips without OA, a significant increase in ΔR1 was observed in the anterosuperior to superoposterior and posterior (*p* = 0.012 ~ 0.042) sub-regions in hips with mild OA and in all sub-regions (*p* = 0.001 ~ 0.020) in hips with moderate to severe OA (Figure 2C).

### Correlation analyses

**Figure 3:**
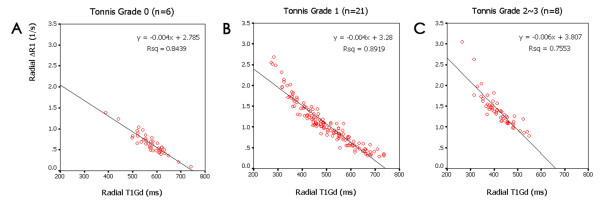
**Scatterplot of** Δ**R1 and corresponding radial T1**_**Gd**_**.** For all 3 subgroups, Tönnis grade 0 (**A**), Tönnis grade 1 (**B**) and Tönnis grade 2~3 (**C**), high correlations (r = −0.869 ~ −0.944, *p* < 0.001) indicated that T1_Gd_ and ΔR1 are equally effective in evaluating cartilage status. The lines represent the linear regression fit.

A high linear correlation was detected between radial T1_Gd_ and ΔR1 in all 3 subgroups. The Pearson correlation coefficient between radial T1_Gd_ and ΔR1 was −0.919 (*p* < 0.001) in hips without OA (Figure 3A), -0.944 (*p* < 0.001) in hips with mild OA (Figure 3 B), and −0.869 (*p* < 0.001) in hips with moderate to severe OA (Figure 3 C).

## Discussion

In this study, different patterns of T1_Gd_ were shown in various radial sub-regions of dyplastic hip joints in pace with the aggravation of secondary OA. In studies performed on healthy asymptomatic volunteers, T1_Gd_ values were reported as 570 ± 90 ms on coronal T1 mapping by Kim et al. [12], and the mean value of T1_Gd_ ranged from 553.9 to 629.4 ms on radial T1 mapping according to Bittersohl et al. [25]. Using the values of healthy asymptomatic volunteers as the normal standard [12,25], hips without radiographic OA in the present study showed a normal mean value of T1_Gd_ (538 ~ 624 ms) in the radial sub-regions from anterior to posterosuperior and a mildly decreased T1_Gd_ value (mean, 502 ms) in the posterior, with a higher T1_Gd_ value toward the superior sub-regions. In hips with mild radiographic OA a significant decrease in T1_Gd_ was detected in the radial sub-regions from anterosuperior to superior, compared to the hips without radiographic OA. In the hips with moderate to severe radiographic OA, a significant overall decrease in T1_Gd_ was found compared to those without radiographic OA. The patterns of cartilage degeneration in this series accorded well with the general understanding of cartilage damage in the hip with dysplasia. Previous arthroscopic and radiographic studies have indicated that there is a high prevalence of cartilage lesions in the anterosuperior and superoanterior regions [26,27], and early degenerative processes in dysplastic hips originate at the watershed zone between the acetabular labrum and the acetabular cartilage [28]. Recently, another study using radial dGEMRIC to evaluate cartilage degeneration in hip dysplasia was published by Domayer et al. [14]. Radial dGEMRIC showed an increased T1 in the weight-bearing areas of the acetabulum when the dGEMRIC index was more than 500 ms and a globally decreased T1 when the dGEMRIC index was less than 500 ms. Those findings corresponded to ours in the subgroups without radiographic OA and with moderate to severe radiographic OA.

The decrease of T1_Gd_ value in the posterior sub-region in hips without OA and with mild OA detected in the current study is not an unexpected finding. Knowledge of normal variations of GAG content within different regions of the hip joint is critical for defining the “abnormal” T1_Gd_ and should be taken into consideration. GAG content was revealed to be higher in the weight-bearing portions of the hip joint with a gradual decrease toward the inferior regions [29,30]. Correspondingly, radial T1_Gd_ shows a zonal variation in adult asymptomatic hip joints, with higher values toward the superior regions and the lowest value noted in the posterior [25]. Further efforts are needed to establish the ideal zonal cutoff value of T1_Gd_ for distinguishing abnormal cartilage from normal.

Whether T1_Gd_ gives information comparable to ΔR1 is one of the central issues in dGEMRIC. Watanabe et al. showed that ΔR1 had a better correlation with biopsy-determined GAG content in transplanted cartilage than either T1_pre_ or T1_Gd_ alone [16]. In native articular cartilage, ΔR1 was equally effective as T1_Gd_ for both OA and healthy subjects [17-19]. According to the study performed on FAI patients by Bittersohl et al., the correlation between ΔR1 and T1_Gd_ within the weight-bearing region of the hip joint was −0.95 in a study cohort with Tönnis grade 0, -0.89 in Tönnis grade 1 and −0.88 in asymptomatic volunteers [19]. Li et al. compared the ability of ΔR1 and T1_Gd_ to differentiate patients with knee OA from healthy subjects [17]. In their study, ΔR1 and T1_Gd_ were found to be highly correlated (r = −0.96) and almost identical in terms of effect sizes and areas under receiver operating characteristic curves. Williams et al. also reported a high correlation (r = −0.87 ~ −0.96) between ΔR1 and T1_Gd_ in knee joints with and without symptoms [18]. Another study found that T1_pre_ values were only minimally different in early cartilage degeneration [31]. Our study showed good consistence with those previous studies. No significant difference of T1_pre_ was detected in any radial sub-regions except the superoanterior and posterior in hips with radiographic OA, compared to those without. Radial ΔR1 was observed to have similar patterns of radial distribution and a high inverse linear correlation with radial T1_Gd_ (r = −0.869 ~ −0.944) in all 3 subgroups. Correlation coefficients between ΔR1 and T1_Gd_ noted by the present study were in accordance with those (r = −0.87 ~ −0.96) noted by previous studies [17-19]. Considering the logistical costs in terms of time and effort to acquire T1_pre_ measurements, the results of the present study support that the current practice of measuring T1_Gd_ is adequate for assessing native cartilage in AD patients using radial dGEMRIC.

The radial dGEMRIC index, with the potential to reflect cartilage status in the entire hip joint, should be effective and helpful for preoperative evaluation in adult AD patients. The different patterns of T1_Gd_ in various radial sub-regions shown in this current study indicate that the articular cartilage from anterior to superior and posterior is vulnerable to degenerative processes, and therefore evaluation of cartilage status in these sub-regions is critical for predicting the postoperative effects in cases with early stages of secondary OA. In contrast, significant overall cartilage damage with advanced OA presages a poor postoperative outcome.

The main limitation of the present study was the lack of a healthy control group, for ethical reasons, and therefore the normal zonal variations of dGEMRIC indices are not available. It may not be accurate enough to define “abnormal” radial T1_Gd_ using the reported normal range of T1_Gd_ value measured on coronal T1 mapping as the normal standard, particularly for the inferior sub-regions. For the same reason, subgroup comparisons were only performed between hips with radiographic OA and those without instead of healthy asymptomatic hips. It is possible that the decrease in T1_Gd_ in hips with radiographic OA was underestimated. However, values of radial T1_Gd_ in hips without radiographic OA in this study were found to be comparable with those in healthy volunteers reported by a previous study [25]. So we believe the issue should have a minimal impact on our conclusion on subgroup comparisons. Future studies should involve healthy asymptomatic volunteers to establish the normal range of radial T1_Gd_ and the best cutoff values for distinguishing abnormal cartilage from normal. In addition, the present study included a relatively inhomogeneous cohort with wide age range (14 to 54 years) and therefore, age related primary OA may be the potential cofounding. Statistically, bilateral observations in part of the study samples may be not strict although no significant correlations (*p* = 0.122 ~ 0.994) of the radial indices were detected between two hips in the same patient. As in previous studies as described by Kim et al. and Bittersohl et al. [12,25], differentiation between acetabular and femoral cartilage was not possible for the ROI analysis on a 1.5 T scanner because of the limitation of spatial resolution. Joint fluid as well as Gd-DTPA^2-^ in the synovial fluid may have altered the T1 value.

## Conclusions

Different patterns of cartilage degeneration were detected in dysplastic hips at different stages of secondary OA by radial dGEMRIC indices, with a significant decline of cartilage function in the radial sub-regions from anterosuperior to superior in those with mild radiographic OA, and overall cartilage damage in those with moderate to severe radiographic OA. Because of the high correlations between ΔR1 and T1_Gd_, pre-contrast measurements seem unnecessary for radial dGEMRIC regardless of the severity of OA. With the ability to reflect cartilage status in the entire hip joint, radial dGEMRIC seems to be a more useful technique than conventional coronal imaging for preoperative evaluation in AD patients at an early stage of secondary OA.

## Abbreviations

OA: Osteoarthritis; AD: Acetabular dysplasia; MRI: Magnetic resonance imaging; dGEMRIC: Delayed gadolinium-enhanced magnetic resonance imaging of cartilage; Gd-DTPA^2-^: Gadopentetate; GAGs: Glycosaminoglycans; 3D: 3-dimensional; FAI: Femoroacetabular impingement; T1_Gd_: Post-contrast T1 relaxation time; T1_pre_: Pre-contrast T1 relaxation time; ΔR1: Difference between the relaxation rates; LCE angle: Lateral center-edge angle of Wiberg; WOMAC: Western Ontario and McMaster Universities Osteoarthritis; a: anterior; as: anterosuperior; sa: superoanterior; s: superior; sp: superoposterior; ps: posterosuperior; p: posterior; ROI: region of interest; ICC: Intra-class correlation; CI: Confidence intervals.

## Competing interests

Ali Guermazi is the President of Boston Imaging Core Lab, LLC, and is a Consultant to Merck Serono, Genzyme, Stryker, AstraZeneca, and Novartis. Yongming Dai is employed by Siemens Healthcare. No other authors have any financial disclosures.

## Authors’ contributions

All authors contributed substantially to drafting and revising the intellectual contents of the manuscript and approved the final version for submission: study design, K-PK, JZ and LX; data collection and magnetic resonance images measurement, LX and YS; data analysis, LX and DH; writing of the initial draft of the manuscript, LX, DH, AG, and XC; technical supporting, YD; guarantor of the integrity of the study: XC. All authors read and approved the final manuscript.

## Pre-publication history

The pre-publication history for this paper can be accessed here:

http://www.biomedcentral.com/1471-2474/13/212/prepub
